# Leukocyte Trafficking and Regulation of Murine Hematopoietic Stem Cells and Their Niches

**DOI:** 10.3389/fimmu.2019.00387

**Published:** 2019-03-05

**Authors:** Daniel Lucas

**Affiliations:** ^1^Division of Experimental Hematology and Cancer Biology, Cincinnati Children's Hospital Medical Center, Cincinnati, OH, United States; ^2^Department of Pediatrics, University of Cincinnati College of Medicine, Cincinnati, OH, United States

**Keywords:** hematopoietic stem cell, niches, leukocyte trafficking, neutrophils, Tregs

## Abstract

Hematopoietic stem cells (HSC) are the most powerful type of adult stem cell found in the body. Hematopoietic stem cells are multipotent and capable of giving rise to all other types of hematopoietic cells found in the organism. A single HSC is capable of regenerating a functional hematopoietic system when transplanted into a recipient. Hematopoietic stem cells reside in the bone marrow in specific multicellular structures called niches. These niches are indispensable for maintaining and regulating HSC numbers and function. It has become increasingly clearer that HSC and their niches can also be regulated by migrating leukocytes. Here we will discuss the composition of murine bone marrow niches and how HSC and their niches are regulated by different types of leukocytes that traffic between the periphery and the niche. Unless otherwise indicated all the studies discussed below were performed in mouse models.

## Organization of the Murine HSC Niche

**Figure 1:**
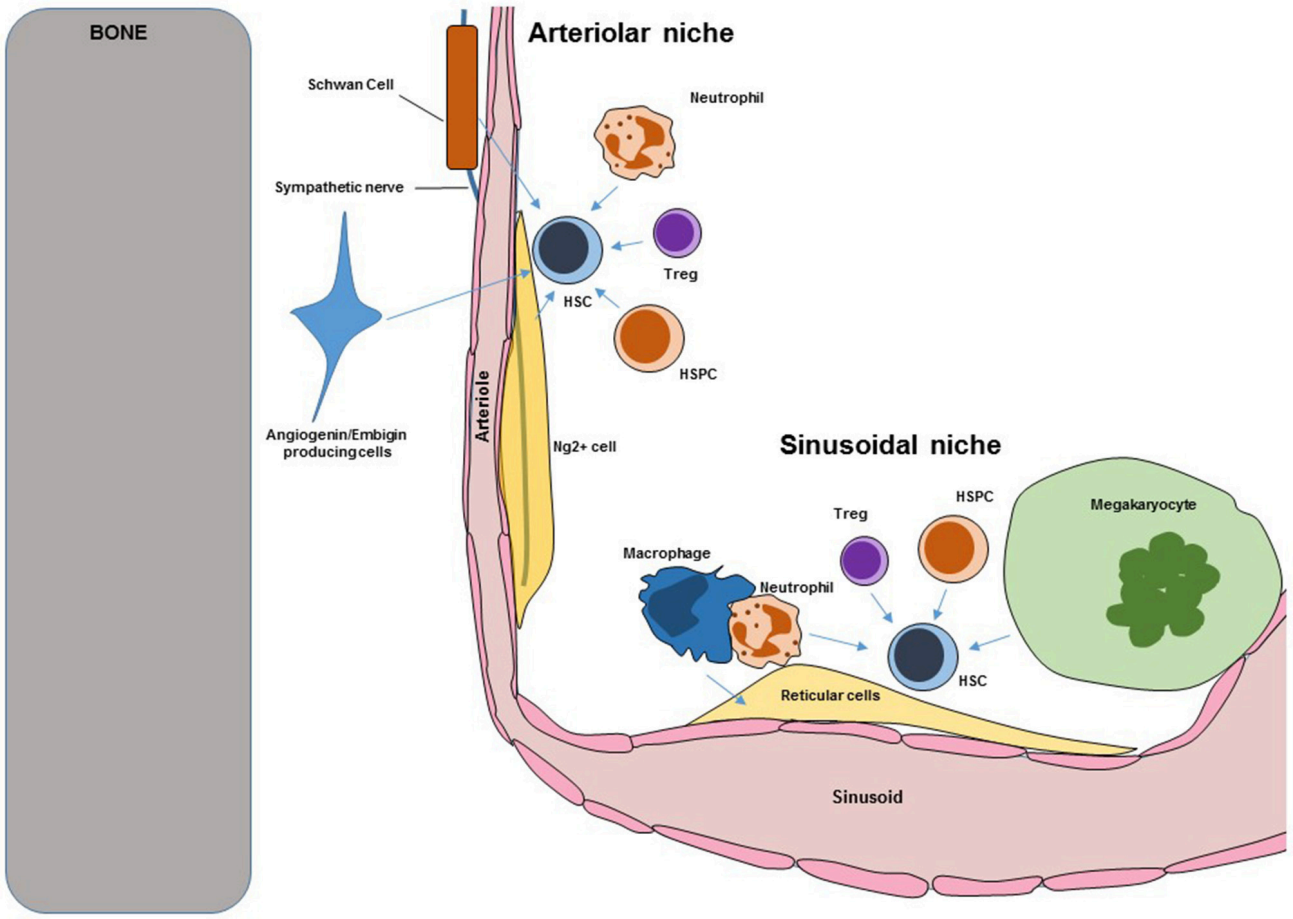
Scheme showing key components of murine BM HSC niches. Blue arrows indicate regulation. HSC, hematopoietic stem cells; HSPC, hematopoietic stem and progenitor cells.

Bone marrow (BM) Hematopoietic stem cells niches are very complex structures in which different cell types with overlapping and unique functions cooperate to regulate HSC maintenance, self-renewal, trafficking, and differentiation. Loss of niche cells or niche-derived signals inevitably leads to loss of HSC. A scheme showing the overall structure of the murine BM niche is shown in Figure 1. Key niche components are:

### Endothelial Cells

The BM is enclosed by bone but blood vessels are the main structure that defines and organizes the BM cavity. Arterioles enter the BM through the bone before giving rise to a dense sinusoidal network that drains through a central vein (1). Imaging studies have revealed that all HSC are intimately associated with the vasculature. Multiple independent approaches confirmed the role of endothelial cells as critical components of the murine niche. The cytokines Cxcl12 and stem cell factor (Scf) are key regulators of HSC trafficking and self-renewal. The Morrison and Link's groups demonstrated that conditional *in vivo* deletion of Cxcl12 or Scf in BM endothelial cells (using *Tie2-cre* mice) was sufficient to cause loss of HSC (2–4). Similarly the Butler group showed that conditional *Jagged1 in vivo* deletion in endothelial cells (using *Ve-cadherin-cre* mice) led to HSC exhaustion (5). In the BM E-selectin is only expressed by endothelial cells (6), the Levesque group showed that HSC in E-selectin knockout mice have reduced cell cycling indicating that endothelial E-selectin promoted HSC proliferation (6). These studies formally demonstrated that endothelial cells are bona-fide niche cells with different functions in regulating steady-state HSC. In addition *in vivo* conditional overexpression of the Notch1 intracellular domain (that leads to increased Notch signaling) in endothelial cells led to increased angiogenesis and HSC numbers (7). In contrast, conditional deletion of *Rbpj* (which is required for transcription of Notch regulated genes) led to deficits in BM endothelial cells (8). These indicate that endothelial cells not only regulate HSC directly but also regulate the number of niches.

The most dramatic example of the importance of endothelial cells in hematopoiesis is during regeneration. Myeloablation (the chemotherapy and/or radiation treatments used to condition the BM prior HSC transplantation) also leads to the almost complete disappearance of BM sinusoids (1, 9). Restoration of a functional sinusoidal network is the limiting step in reestablishing normal hematopoiesis (9). This is not only due to restoration of their homeostatic functions but because endothelial cells upregulate molecules like Jagged2 and Pleiotrophin that promote HSC engraftment and hematopoietic regeneration (10, 11). The precise mechanisms through which sinusoids are restored are not well established although it is known that the *Vegf, Tnfa, Nf*κ*B*, and *Angiopoietin1* pathways have different contributions during regeneration (9, 12–14).

### Stromal Cells

#### Reticular Stromal Cells

The BM is crisscrossed by a network of reticular stromal cells that also associates with blood vessels. These stromal cells produce Cxcl12, Scf, Pleiotrophin, and other cytokines that maintain and regulate HSC (15). These cells receive different names depending on the method used to isolate them. For example CAR stands for Cxcl12-abundant reticular cells and are isolated using *Cxcl12-gfp* reporter mice, *Nestin-GFP*^*dim*^ cells are isolated using *Nestin-gfp* reporter mice and LepR^+^ cells are detected using *LepR-cre:Tomato* mice (15). Because the *LepR-cre* mouse also allows genetic manipulation of the reticular stromal cells it is quickly becoming the method of choice to label these cells (3). Reticular cells have osteoprogenitor and adipogenic potential *in vivo*, have mesenchymal stem cell activity (i.e., can differentiate to osteoblasts, adipocytes, and chondrocytes) *in vitro*, and upon transplantation in ossicles can generate an ectopic niche that supports extramedullary hematopoiesis (16, 17). Conditional deletion of *Cxcl12, Scf*, *Pleiotrophin* and other genes in these reticular cells results in loss of HSC (2–4, 11). Taken together these studies formally demonstrate that reticular stromal cells are a niche for HSC in the BM.

#### Periarteriolar Ng2 ^+^ Stromal Cells

These are an extremely rare population of stromal cells that ensheathes the arterioles in the BM. These cells can be labeled as Nestin-GFP^bright^ cells using *nestin-gfp* mice or as Ng2^+^ cells using *Ng2-cre*^*ERT*2^*:gfp mice* (1). Even though they are very rare they are key regulators of HSC function. Imaging analyses showed that ~30% of BM HSC localized to arterioles and that this association was closer than expected from random suggesting that these cells might be niche components. Conditional depletion of Ng2^+^ cells using *Ng2-Cre*^*ERT*2^*:iDTR* mice led to HSC loss (1). In a follow up experiment the Frenette group showed that Ng2^+^ cells are the major source of CXCL12 in the BM and that conditional *Cxcl12* deletion in these cells led to loss of quiescent HSC (18). These results demonstrate that Ng2^+^ periarteriolar stromal cells are a key component of the HSC niche.

#### Non-myelinating Schwann Cells

These are very rare cells that ensheath the sympathetic nerves that enter the BM via arterioles. Because of this they are intimately associated with arterioles and HSC. In a seminal study the Nakauchi lab showed that these glia cells are the main source of activated Tgfβ in the BM and that sympathetic denervation led to the loss of these cells and concomitant HSC loss (19). Despite their role in HSC maintenance the function and regulation of these cells is not well-studied. This is because it has not been yet possible to purify these cells for more detailed analyses.

#### Embigin and Angiogenin-Producing Cells

A fraction of HSC localizes close to the endosteal surface of the bone. By purifying and comparing the expression profile of endosteal cells that were proximal or distal to HSC after transplantation the Scadden and Hu laboratories identified embigin and angiogenin as candidate HSC “niche” factors. Conditional angiogenin deletion in reticular stromal cells and Ng2^+^ periarteriolar cells led to increased numbers of HSC in the BM due to increased proliferation but these HSC were deficient in engraftment potential indicating that angiogenin is necessary to maintain HSC function (20). In addition they found that angiogenin deletion in Osterix^+^ osteoprogenitors also caused loss of HSC indicating that these cells also function as an HSC niche (20). The mechanism of action of angiogenin is especially interesting. Angiogenin is a ribonuclease secreted by stromal cells that is then imported into HSC where it modulates endogenous RNAs. This causes reductions in protein synthesis and increases in HSC function (21). Antibody blockade of embigin leads to HSC proliferation and accumulation in the BM. Embigin producing cells could be isolated as col2.3-GFP^+^Embigin^+^VCAM^+^ cells (using *col2.3-gfp* reporter mice) and were also enriched for CXCL12. These experiments demonstrated that these cells are a novel component of the niche (20).

### Hematopoietic Cells

#### Megakaryocytes

These are very large multinucleated cells that localize, exclusively, to the sinusoids where they release platelets to the circulation. They are hematopoietic cells and were the first hematopoietic cells shown to directly regulate HSC. The role of megakaryocytes in the niche was independently discovered by the Frenette', Li', and Suda's groups (22–25). Imaging analyses revealed that most sinusoidal HSC are also in contact or within 5 μm of megakaryocytes. Megakaryocyte depletion using *Cxcl4-cre:iDTR* or *Cxcl4-cre:Mos-iCsp3* mice induced a 10-fold increase in BM HSC due to hyperproliferation that was followed by HSC loss due to exhaustion (22–25). These results indicated that megakaryocytes maintain HSC numbers and fitness by restricting proliferation. Megakaryocytes are the main source of the cytokine CXCL4 and *Cxcl4*^−/−^ mice had fewer functional HSC (22). Megakaryocytes are also a major source of TGFβ and administration of these cytokine into megakaryocyte-depleted mice rescued the HSC phenotype (25). Bone marrow megakaryocytes also produce thrombopoietin which is known to regulate HSC quiescence. Deletion of the C-type lectin like receptor-2 in megakaryocytes using *Cxcl4-cre:Clec2*^*flox*/*flox*^ mice led to impaired thrombopoietin production by megakaryocytes and fewer megakaryocyte and HSC numbers (23, 24). These results indicate that megakaryocytes regulate HSC numbers and function by secreting Cxcl4, Tgfβ, and Thrombopoietin (22–25).

#### Hematopoietic Progenitors

Hematopoietic stem cells and progenitors can also regulate each other. Because E-selectin induces HSC proliferation (6) the Hidalgo lab examined whether expression of the E selectin ligand Esl1 in HSC might mediate this regulation. Unexpectedly they found reduced HSC numbers and proliferation in Esl1-knockout mice. Further when Esl1-deficient hematopoietic stem and progenitor cells (HSPC) were cotransplanted together with WT HSPC into WT recipients the WT HSPC also showed reduced numbers and proliferation. These indicated that Esl1 expression regulated HSPC proliferation in a non-autonomous manner (26). This is likely mediated via two different effects. The first one is the observation that hematopoietic Esl1 deficiency leads to reductions in a key component of the murine niche: reticular stromal cells (26). The second is modulation of Tgfβ activity as blockade of this pathway in cocultures of WT and Esl1-deficient HSPC rescued the proliferation defect in both genotypes (26). This study shows that HSC and their immediate offspring regulate each other and reticular stromal cells in the niche.

#### Leukocytes

These are mature hematopoietic cells that were thought to have no role in regulating hematopoiesis. However, a series of studies in the last decade have demonstrated that mature leukocytes (macrophages, neutrophils, and T-cells) are critical regulators of HSC and niche function and that leukocyte trafficking also impacts HSC. How migrating leukocytes function in the niche is the focus of the second part of the review.

### Other Candidate Niche Cells

In addition to the cell types described above, other stromal cells (osteoblasts, osteocytes, osteoclasts, and adipocytes) have been proposed to be components of the HSC niche in some studies while other studies have shown no role for these cells in HSC regulation. The evidence for and against the role of each of these cells in the niche was reviewed recently (27). Additional studies are needed to precisely clarify the role of these cells in the HSC niche and in the BM microenvironment.

### Distal Regulation of Bone Marrow HSC by Other Organs

All the cell types described above reside in the BM and most of them are intimately associated with HSC. Imaging of HSC location and interaction with candidate niche cells remains one of the most powerful tools to identify new components of the niche. However, an emerging concept in the field is that HSC and their niches can be regulated (directly or indirectly), long-distance, by different organs.

The nervous system is the best characterized organ(s) that regulates HSC distally. The initial discovery showed that sympathetic innervation of the BM is necessary for HSC release from their niches into the circulation (28). Follow up studies showed that the sympathetic nervous system orchestrates daily oscillations of Cxcl12 production by the niche and thus controls HSC trafficking (16, 29), regulates the regeneration of the niche after myeloablation (30) and even controls niche remodeling during hematopoietic malignancies (31, 32) and aging (33).

HSC can also be regulated by hormones; parathyroid hormone acts on stromal cells (likely reticular stromal cells) increasing their number and thus leading to increased HSC numbers (34). In female mice, estrogen acts directly on HSC to drive their proliferation (35). Ovariectomy suppressed this effect indicating that ovaries are the source of estrogen that regulates HSC (35). Pituitary glucocorticoids act directly on HSC to impair their mobilization in response to granulocyte colony-stimulating factor (G-CSF) (36). A series of studies in zebrafish and mouse models and with human cells showed that prostaglandins positively regulate HSC numbers under homeostasis and can be used to promote regeneration and HSC engraftment after *in vivo* and *ex vivo* treatments and to mobilize stem cells from the bone marrow to the circulation where they can be harvested for transplantation (37–41).

Two recent studies demonstrated that the liver and the intestine also regulate BM HSC. Thrombopoietin has long been known to regulate HSC quiescence but the source of this cytokine remained unknown. Using elegant conditional deletion experiments, the Ding lab suggested that BM sources of thrombopoietin did not regulate HSC (42). Instead they found that deletion of thrombopoietin from hepatocytes results in loss of HSC quiescence and subsequent exhaustion (42). Although very interesting, this study raises two important questions. The first one is that megakaryocyte-derived thrombopoietin was reported to regulate HSC quiescence (23, 24). The second is that thrombopoietin is also required for megakaryocyte maturation and megakaryocytes regulate HSC quiescence (22–25). It will be interesting to dissect the contribution of megakaryocyte- and hepatocyte-derived thrombopoietin to HSC maintenance and to determine whether they function by acting directly on HSC or indirectly by regulating megakaryocyte numbers. The intestine also regulates BM HSC distally; the Hidalgo lab showed that intestinal macrophages regulate the activity of the niche by modulating G-CSF production (43). Because these macrophages are regulated by trafficking leukocytes their function and regulation are discussed in detail in the next section.

## Functional and Spatial Heterogeneity in the Niche

In the last 5 years it has become increasingly clear that HSC are not a homogeneous population and can be fractionated (based on expression of different markers) into subsets with different *in vivo* potential. Examples of this heterogeneity are the use of *Hdc-GFP* reporter mice to identify Hdc-GFP^+^ myeloid biased HSC (44); the use of von Willebrand factor-reporter mice to identify HSC biased toward megakaryocyte production (45); different levels of reactive oxygen species (ROS) (46); and differences in cell cycle status (1). The mechanisms underlying this heterogeneity are not known but it is likely that this is mediated by interactions with components of the niche in arteriolar and sinusoidal locations. Several lines of evidence support this. Ng2^+^ cells localize, exclusively, to arterioles where they are intimately connected with endothelial cells, sympathetic axons, and GFAP^+^ Schawnn cells (1). In contrast, sinusoids are surrounded by a network of reticular cells and are the site where megakaryocytes localize (3, 22). Imaging analyses (defining HSC as Lin^−^CD48^−^CD41^−^CD150^+^ cells) showed that most HSC associate with sinusoids with a smaller fraction that localizes close to arterioles (1, 46). The large majority of BM HSC (80%) are quiescent with a smaller fraction (20% of all HSC) actively cycling. Using Ki67 to detect cycling and non-cycling HSC the Frenette lab found a statistically significant difference in the localization of Ki67^+^ and Ki67^−^ HSC with the latter group localizing farther away from arterioles (1). Ablation of Ng2^+^ periarteriolar cells using *Ng2-cre*^*ERTM*^*:iDTR* mice lead to reductions in HSC numbers, loss of quiescence, and relocalization of HSC away from arteries (1). These results suggest that arterioles are a niche that maintains a subset of HSC that associate with them, and that subset is enriched in quiescent HSC. In agreement with these results the Lapidot lab showed that HSC could be fractionated according to the level of reactive oxygen species (ROS) by *in vivo* injection of hydroethidine (46). Previous studies had shown that ROS cause HSC proliferation and migration in the bone marrow (47, 48). The Lapidot lab found that HSC that localized to arterioles were uniformly ROS^low^ (and presumably quiescent) whereas HSC that localized to sinusoids could be ROS^high^ or ROS^low^ (46). They also found that conditional deletion of *Fgfr1* and *Fgfr2* in endothelial cells caused increased vascular permeability which in turn caused ROS accumulation in the stem cells and reductions in HSC numbers. This was due to the increases in ROS levels as treatment with a ROS scavenger rescued the HSC defect in the *Fgfr1/2* conditional knockouts (46). In agreement with this, the Frenette group recently reported that conditional SCF deletion in arteriolar, but not sinusoidal, endothelial cells (using *Bmx1-cre* as an arteriole-specific cre) caused HSC loss (49).

The studies above support the concept that arterioles maintain a subset of HSC that is enriched for cells in a quiescent/low metabolic status. However, sinusoids also maintain a subset of HSC while promoting quiescence. Megakaryocytes localize to the sinusoids and ROS^low^ HSC in the sinusoids colocalize with megakaryocytes (46): loss of megakaryocytes or megakaryocyte-derived molecules like Cxcl4, Tgfβ, and Thrombopoietin lead to HSC proliferation and exhaustion (22–25). However, megakaryocyte ablation did not impact the localization of the HSC subset close to arterioles suggesting that arteriolar and sinusoidal niches were functionally independent (22). The Jacobsen and Nerlov labs showed that von Willebrand factor-reporter mice can be used to identify vWF-eGFP^+^ HSC that were biased toward a myeloid and megakaryocytic fate whereas vWF-eGFP- HSC were lymphoid biased. Using imaging analyses the Frenette lab reported that vWF-eGFP^+^ HSC localized to sinusoidal megakaryocytes whereas vWF-eGFP^−^ HSC localized to arterioles (50). Megakaryocyte depletion using *CD169:iDTR* mice caused exhaustion of myeloid-biased vWF-eGFP^+^ HSC but not lymphoid-biased vWF-eGFP^−^ HSC (50). Ng2^+^ cell depletion using *NG2-cre*^*ERTM*^:*iDTR* mice caused loss of lymphoid-biased but not myeloid-biased HSC (50).

Arteriolar endothelial cells, Schawnn cells, sympathetic nerves, and Ng2^+^ periarteriolar cells are intimately associated (1). Similarly, sinusoidal endothelial cells are tightly associated with reticular stromal cells and megakaryocytes (3, 22). These suggest that different types of niche components associate form spatially and functionally independent niches that maintain different subsets of HSC by promoting quiescence. They also suggest that the subset of cycling/metabolically active HSC (20% of all HSC) localizes to the sinusoids where they are maintained in a megakaryocyte-independent manner. However, controversies remain. For example the Frenette group recently reported that Cxcl12 deletion in Ng2^+^ periarteriolar cells but not LepR^+^ reticular stromal cells caused BM HSC loss (18). This challenges two manuscripts by the Morrison's and Link's groups showing that reticular stromal cells are the major source of Cxcl12 that maintains HSC numbers (2, 4). Another controversy is that while imaging analyses using Lin^−^CD48^−^CD41^−^CD150^+^ to identify HSC showed a clear association between a subset of HSC and arterioles (1, 46) imaging HSC as α-catulin-GFP^+^c-kit^+^ cells in α*-catulin-gfp* reporter mice did not find a specific association of HSC with arterioles (51). Instead they found that all HSC preferentially associated with sinusoids and LepR^+^ perivascular cells (51). Thus, more detailed analyses are needed to reconcile these results.

## Regulation of HSC and Their Niches by Leukocytes. Role of Leukocyte Trafficking

**Figure 2:**
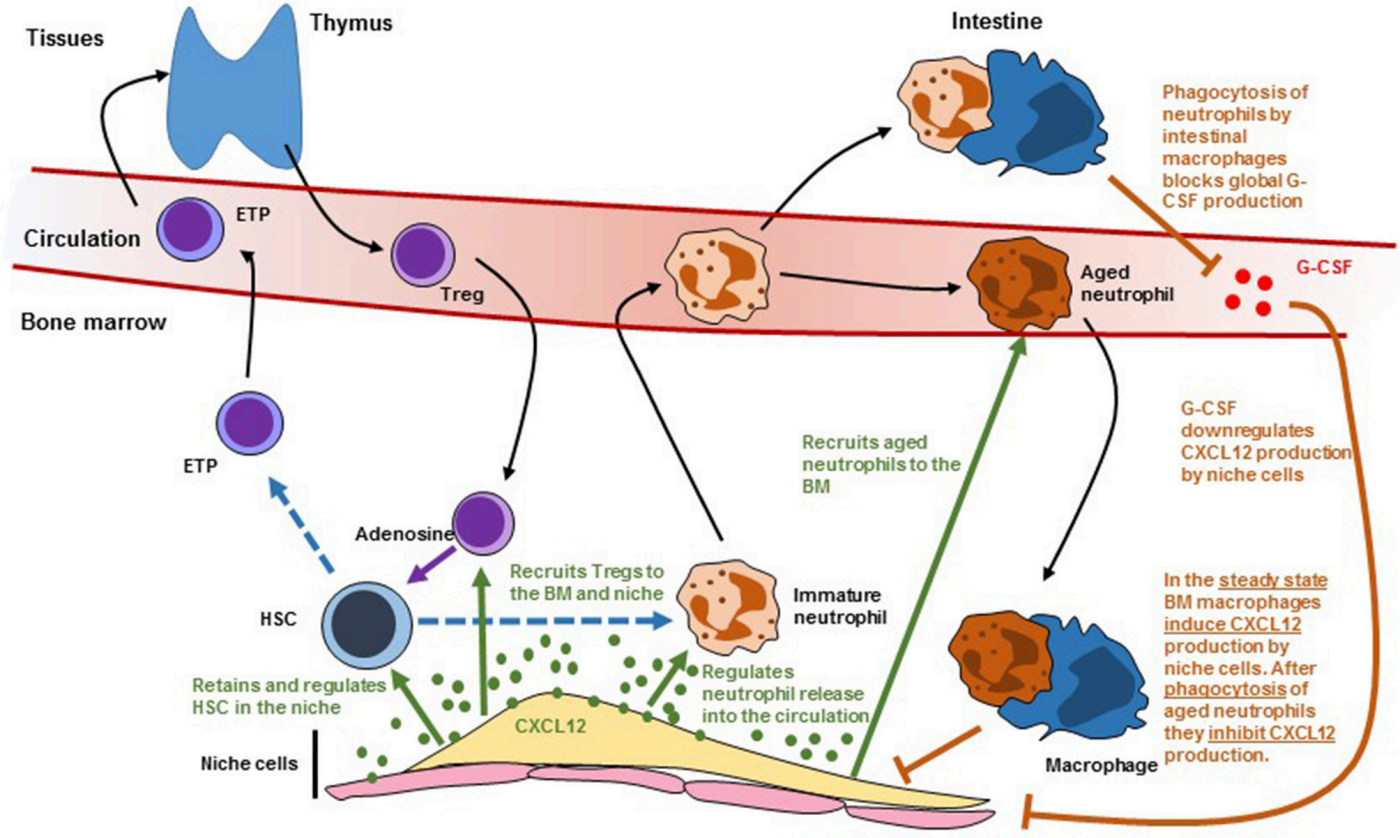
Scheme showing how leukocyte trafficking regulates (and is regulated by) niche cells. Dashed lines indicate differentiation. Solid black arrows indicate migration. Solid green arrows indicate regulation by CXCL12. Solid orange arrows indicate regulation by macrophages. HSC, hematopoietic stem cells**;** ETP, early T cell progenitor; G-CSF, granulocyte colony-stimulating factor.

HSC and their niches are also regulated by different types of leukocytes. This adds two layers of complexity to HSC regulation. All leukocytes are the offspring of stem and progenitor cells. When leukocytes impact the number and function of the HSC they will, ultimately, affect their own production which in turn might further affect HSC function. This is further complicated because many of the pathways that regulate HSC retention in the niche and trafficking into the circulation like Cxcl12 (Figure 2), S1P (52), Ccr2 (53), and Cxcr2 (54) signaling also regulate leukocyte trafficking. In this section we discuss recent advances showing how leukocytes regulate HSC and their niches and how leukocyte migration impacts these regulatory mechanisms.

### Macrophages

Three independent studies showed a critical role for BM macrophages in retaining HSC in the niche. While studying the mechanisms of G-CSF-induced HSC mobilization the Levesque lab noticed that G-CSF caused loss of a population of macrophages that was intimately associated with endosteal cells and that they named “osteomacs.” Selective depletion of myeloid cells using MAFIA mice or phagocyte depletion with clodronate-loaded liposomes caused osteomac loss and HSC mobilization. This correlated with downregulation of Cxcl12 and SCF in stromal cells purified from the endosteum (55). The Link laboratory had previously shown that G-CSF acts on a hematopoietic cell to induce mobilization but the identity of these cells remained unknown (56). In a follow up study they generated *CD68-G-CSFR* mice in which the G-CSF receptor is expressed, exclusively, in monocytes and macrophages (57). G-CSF-induced HSC mobilization in this model was as potent as in wild-type mice and caused loss of monocytes/macrophages in the BM. Since G-CSF functions by downregulating Cxcl12 in the niche these experiments demonstrated that monocytes/macrophages modulated niche activity in response to G-CSF (57). The Frenette lab hypothesized that BM macrophages might regulate Cxcl12-producing reticular cells in the niche. Using three models of monocyte/macrophage ablation (*CD11b-DTR*, MAFIA and clodronate-loaded liposomes) they demonstrated that monocyte/macrophage ablation was sufficient to downregulate *Cxcl12* production in BM reticular stromal cells. They then used *CD169-DTR* mice, which depletes macrophages but not monocytes, to demonstrate that macrophages controlled niche function (58). These studies showed that macrophages crosstalk with niche components to control the production of Cxcl12 that retains HSC in the niche. It is also possible that macrophages might regulate HSC directly. The Lapidot lab found rare αSMA1^+^COX2^+^ macrophages that colocalized with HSC. These cells could promote HSC survival *in vitro* in a COX2-dependent manner (likely via prostaglandin E2 production by COX2). *In vivo* pharmaceutical COX2 inhibition led to HSC depletion suggesting that these αSMA1^+^ macrophages maintain HSC (59).

Macrophages also modulate HSC engraftment after transplantation. Kaur et al. used *Csf1r-gfp* reporter mice to demonstrate that CD169^+^ BM macrophages survived irradiation and regenerated autonomously (i.e., independently of donor HSC). Depletion of these macrophages using *CD169-DTR* mice completely blocked HSC engraftment demonstrating a critical role for macrophages during regeneration (60).

### Neutrophils

These are short-lived cells that are indispensable to maintain innate immunity. They are produced in large numbers in the BM and enter the circulation and tissues before being cleared a few hours later in the BM, liver, and spleen. The Hidalgo lab found that aged neutrophils can be defined as CD62L^lo^Cxcr4^hi^ cells and discovered that the number of these aged neutrophils in the blood oscillated following a circadian pattern (61). Because previous studies showed that circadian oscillations in sympathetic activity regulated Cxcl12 production in the niche (16) they investigated whether neutrophils could also impact the niche. They found that circadian oscillations in the number of Cxcl12-producing reticular cells in the BM were controlled by neutrophil trafficking; neutrophil depletion, or blocking recruitment of aged neutrophils to the BM (by deleting Cxcr4 in neutrophils) led to increases in reticular stromal cells and reduced HSC release into the circulation (61). These phenotypes required that BM macrophages phagocytosed the aged neutrophils and was dependent on expression of LXR receptors in the macrophages (61). This study was the first demonstration of a mature cell regulating niche size. In a follow up study the Hidalgo lab found that neutrophil trafficking into the intestine also controlled HSC niche activity in the BM. Mice deficient in FUT7 have neutrophils with limited ability to extravasate into tissues. These mice also showed reduced numbers of Cxcl12-producing reticular niche cells in the bone marrow and constitutive HSC release in the circulation (43). These phenotypes can be rescued by parabiosis (joining the circulation) with WT mice indicating that neutrophil extravasation regulates niche activity (43). Surprisingly, the niche and HSC trafficking phenotype could also be rescued when *Fut7*^−/−^ mice were parabiosed with *Mrp8-cre;Cxcr4*^*fl*/*fl*^ mice in which neutrophil recruitment to the BM is completely abolished (43). This indicated that the niche defect in *Fut7*^−/−^ mice was independent of the recruitment of aged neutrophil to the BM described above (61). When analyzing the fate of extravasated neutrophils they found that only intestinal macrophages failed to engulf *Fut7*^−/−^ neutrophils. They found that neutrophil phagocytosis by intestinal macrophages inhibited IL23 production by these cells; this in turn led to lower global levels of G-CSF which led to reduced HSC release from BM niches (43). In the *Fut7*^−/−^ mice there were increased IL23 and G-CSF levels and antibody blockade of either molecule was sufficient to correct HSC release from the niche (43). These two studies highlight how neutrophil trafficking and phagocytosis by macrophages in different tissues controls niche activity. It is also likely that neutrophils also regulate HSC function directly. Using histidine decarboxylase-GFP (*Hdc-GFP)* reporter mice, the Wang lab found that around 10% of HSC were Hdc-GFP^+^. In transplantation experiments these Hdc-GFP^+^ HSC produced higher numbers of myeloid cells than the Hdc-GFP^−^ HSC indicating that they were myeloid biased (44). They also found that Hdc-GFP^+^ HSC in the marrow of *Hdc*^−/−^ mice cycled faster which in turn led to exhaustion and loss of myeloid biased HSC, indicating that histamine maintains Hdc-GFP^+^ HSC by restricting their proliferation (44). The major source of histamine in the bone marrow are neutrophils and imaging analyses showed that myeloid biased Hdc-GFP^+^ HSC were in contact with HDC-GFP^+^ neutrophils. In contrast there was no specific association between HDdc-GFP^+^ neutrophils and Hdc-GFP^−^ HSC. These results indicate that histamine producing cells (likely neutrophils) regulate myeloid biased HSC (44). A second possibility is that Hdc-GFP^+^ HSC might regulate themselves via histamine secretion in an autocrine loop. Neutrophils also control the regeneration of endothelial cells in the niche. After myeloablation, immature BM neutrophils are recruited to injured vessels where they promote vessel and hematopoietic regeneration via TNFα secretion (12).

### Regulatory T Cells

The Lin laboratory found that, after allogeneic transplantation of HLA-mismatched HSC, the donor stem cells survived in the recipients without any type of immunosuppression indicating that BM niches were immune privileged (62). Imaging analyses showed that these allogeneic HSC were surrounded by Foxp3^+^ regulatory T cells. Depletion of Tregs by using *FoxP3-DTR* mice led to loss of the allogeneic HSC. Transfer of WT but not IL10^−/−^ Tregs prevented allogeneic HSC loss after transplantation (62). These results demonstrated that Tregs confer immune privilege to the niche via IL10 signaling. Tregs also regulate HSC in the steady-state. Tregs in *FoxP3-cre;Cxcr4*^*fl*/*fl*^ mice have reduced trafficking to the BM. These causes a ~2-fold increase in HSC and was mediated by increased reactive oxygen species (ROS) in HSC as antioxidant treatment rescued the HSC expansion (63). Imaging analyses showed that Tregs localized close to *Cxcl12*-producing reticular cells and sinusoids and that a subset of Tregs that expressed high levels of CD150 associated with HSC (63). These CD150^+^ Tregs regulate HSC via adenosine as *FoxP3-cre;CD39*^*fl*/*fl*^ mice, in which Tregs are deficient in adenosine production, or wild-type mice treated with adenosine receptor antagonists, also showed increased HSC numbers (63). These studies show that Treg recruitment to the niche regulates BM HSC metabolism.

## Conclusions and Open Questions

Different types of leukocytes traffic between the periphery and the BM where they regulate the numbers and function of HSC and their niches. These regulatory pathways likely crosstalk at multiple different levels (Figure 2). For example, Treg recruitment to the niche is mediated by Cxcl12/Cxcr4 signaling and LepR^+^ reticular stromal cells (63). Macrophages regulate Cxcl12 production in the niche after being activated by phagocytosing aged neutrophils (61). These neutrophils are also recruited to the BM via Cxcl12/Cxcr4. The same signals regulate immature neutrophil release to the circulation which might impact the ability of intestinal macrophages to regulate systemic G-CSF which will further impact niche function (43). The Cxcl12/Cxcr4 pathway is not the only signal that regulates both HSC and leukocyte trafficking. *Cxcr2*^−/−^ mice have increased HSC numbers but these stem cells have impaired function in transplant assays (54). This study indicated that Cxcr2 signaling regulates HSC. However, Cxcr2 is a critical regulator of neutrophil trafficking and *Cxcr2*^−/−^ neutrophils are retained in the bone marrow (64). This neutrophil trafficking defect presumably will alter niche function through the mechanisms described in the previous section. Sphingosine 1-phosphate (S1P) regulates HSC trafficking from blood to tissues and lymph (52). However, S1P also triggers Cxcl12 release by reticular stromal cells (65) and inhibits Treg differentiation (66). Loss of adhesion molecules like selectins and integrins (α4, β1, or β2) all impact both HSC and leukocyte trafficking. Teasing apart direct effects on stem cells from those mediated indirectly by alterations in leukocyte migration is necessary to gain a better understanding of how these pathways regulate normal and diseased hematopoiesis.

The crosstalk between leukocytes and HSC niches likely functions as a biological rheostat through which the BM monitors the periphery. Leukocyte numbers and trafficking are altered after inflammation or infection, and in many hematological diseases (for example acute myeloid leukemia or myelodysplastic syndromes). It is likely that alterations in leukocyte trafficking direct bone marrow hematopoietic output and contribute to the disease phenotypes. This is an area of great interest for future investigations.

## Author Contributions

The author confirms being the sole contributor of this work and has approved it for publication.

### Conflict of Interest Statement

The author declares that the research was conducted in the absence of any commercial or financial relationships that could be construed as a potential conflict of interest.
